# Physiotherapy training and education prior to elective Caesarean section and its impact on post-natal quality of life: a secondary analysis of a randomized controlled trial

**DOI:** 10.1186/s13104-023-06550-5

**Published:** 2023-10-13

**Authors:** Kalani Weerasinghe, Mohamed Rishard, Subhani Brabaharan, Yasaswi Walpita

**Affiliations:** 1 Health and Wellness Unit, Faculty of Medicine, University of Colombo, Colombo, Sri Lanka; 2 Department of Allied Health Sciences, Faculty of Medicine, University of Colombo, Colombo, Sri Lanka; 3 Department of Obstetrics and Gynecology, Faculty of Medicine, University of Colombo, Colombo, Sri Lanka; 4 National Hospital Sri Lanka, Colombo, Sri Lanka; 5 Department of Community Medicine, Faculty of Medicine, University of Colombo, Colombo, Sri Lanka

**Keywords:** Physiotherapy training, Physiotherapy education, Caesarean section, Enhanced, Post-operative recovery, Post-partum, Post-natal quality of life

## Abstract

**Background:**

Caesarean section (CS) is associated with numerous complications that lead to the delayed return to functional activities that have a negative influence on the post-natal quality of life (QOL). It is evident that providing regular evidence-based physiotherapy training and education prior to elective CS helps to enhance the post-natal QOL by improving physical, mental, social, and general well-being. The purpose of this study was to examine the effectiveness of physiotherapy training and education prior to elective CS on post-natal QOL.

**Methods:**

This single-blind parallel randomized controlled study was carried out at De Soysa Hospital for Women (DSHW), Colombo. The study enrolled 54 women who were scheduled to undergo elective CS. The intervention group (n = 27) of women received physiotherapy training and education, while the control group (n = 27) received standard nursing care. In addition to the primary outcome measures, post-natal QOL was measured. The results were examined using descriptive statistics and the independent samples t-test in IBM SPSS 20.

**Results:**

The intervention group showed a higher post-natal QOL for the domains of physical function, role limitation due to physical health, energy/fatigue, and pain than the control group (p < 0.05).

**Conclusion:**

Physiotherapy training and education prior to elective CS play a pivotal role in improving the physical health-related domains of QOL following CS.

**Trial Registration:**

The Sri Lanka Clinical Trials Registry (https://www.slctr.lk). Registration number: SLCTR/2019/029-APPL/2019/028; Registration date: 6th of September 2019.

## Introduction

Caesarean Section (CS) is one of the most commonly performed surgeries in obstetrics. It involves the delivery of a live or dead fetus through an open abdominal incision (laparotomy) and a uterine incision (hysterectomy) [1]. CS rates increased from 5% to 1970 to 31.9% in 2016 [2]. Despite ongoing efforts to reduce the number of CS, experts do not anticipate a significant drop for at least a decade or two [3]. The post-partum period, which begins after the birth of the child and ends at 42 days, can be associated with complications such as post-partum haemorrhage, deep vein thrombosis, pulmonary embolism, gravitational oedema, puerperal infection, breast-feeding difficulties, postural difficulties, backache, during which women should be monitored [4, 5]. This is associated with many complications that lead to a delayed return to functional activities that have a negative influence on the post-natal quality of life (QOL) [6].

When women experience complications following CS in the post-partum period, it may have a significant impact on their physical, mental, and social well-being [7]. It is evident that providing post-partum care to improve women’s health and QOL is crucial and this necessitates a regular evidence-based exercise program [8]. The study on post-natal exercises and QOL on immediate post-partum mothers by Mahishale and the co-authors suggests that exercises administered during the immediate post-partum period facilitate early mobility and improve the post-natal QOL in the domains of physical, mental, social, and general well-being in women following CS [7].

Pre-natal education plays a crucial role in maternal care, which subsequently enhances post-natal QOL globally [9]. Balasoiu and colleagues conducted a study that revealed women who attended pre-natal education sessions recognized the importance of the topic in shaping outcomes related to pregnancy and post-natal life [10]. They concluded that women who attended the lectures acknowledged the benefits of pre-natal education and followed them, whereas those who did not participate underestimated the utility of the topics (p < 0.001) [10]. In an Iranian study investigating the impact of pre-natal education on mothers’ post-partum QOL, the intervention group exhibited significantly higher scores in QOL from 6 to 8 weeks to 1 year and one year following the child’s birth compared to the control group (p < 0.05) [11]. Kaur and the team assessed the effectiveness of early ambulation in post-operative recovery among post-cesarean mothers. The experimental group was early ambulated at 6 h after CS, covering a distance of 40 m, while the control group followed routine care, ambulating after 13–14 h post-CS [12]. Post-operative recovery and QOL were assessed in both groups using a structured nursing assessment sheet, and it was found that early ambulation improves post-operative QOL [12]. Sampselle et al. also concluded that women who exercised in the post-partum period had benefits such as decreased weight retention and higher post-natal QOL scores [13].

Although post-partum exercises are widely accepted to be implemented during the immediate post-partum period, very few studies have systematically investigated aspects such as optimal timing of physiotherapy education and its impact on enhanced post-natal care globally [14]. Though Sri Lanka has a very good track record in the provision of maternal care services in the antenatal period, the provision of care around the time of the delivery and subsequent period has much to be desired [15]. Post-natal care is a key factor that is often neglected in many settings. Poor quality of care around the time of childbirth is associated with a longer hospital stay, poor maternal satisfaction, increased readmission rates, increased cost and poor reputation of the hospital [16, 17]. In the context of the current economic crisis in Sri Lanka [18], we should aim to improve the quality of care by incorporating simple and cost-effective approaches to minimize complications and enhance recovery.

The study aimed to determine the effectiveness of pre-emptive physiotherapy following elective CS on post-natal QOL. It was hypothesized that a comprehensive physiotherapy program, combining pre-operative training and education, followed by reinforcement in the post-operative period with regular feedback and encouragement, would improve post-natal QOL in them.

## Methods

The CONSORT 2010 statement guidelines regarding randomized trials (www.consortstatement.org) were followed for this [19].

### Trial design

**Fig. 1:**
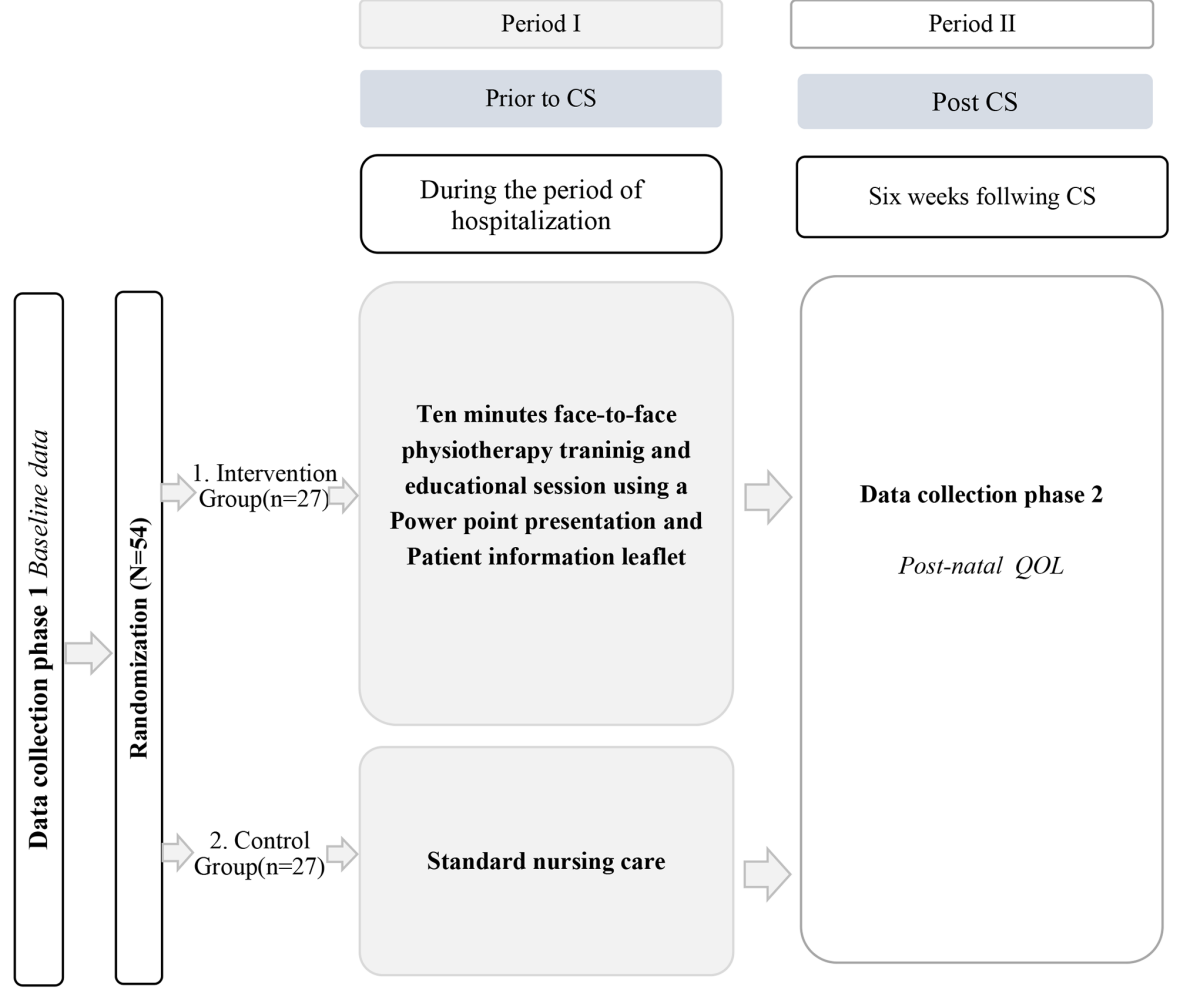
Flow chart of participants

This is a secondary analysis of a RCT that was conducted at De Soysa Hospital for Women (DSHW), Colombo to investigate the impact of antenatal physiotherapy education and training on the post-operative outcomes of women after CS such as post-operative pain, doses of additional analgesics required, pain upon returning to functional activities, and lengths of hospital stay [1]. Figure 1 describes the flow of this secondary analysis.

### Participants

Eligible women aged 20–40 years who were to undergo elective CS were asked to participate in the study. According to the unit policy, all women scheduled for elective CS are admitted to the wards 24 h before the scheduled time. Therefore, women who provided their consent were promptly recruited for the study immediately after their admission, ensuring their inclusion in the trial without delay. This allowed for a standardized and consistent approach in the recruitment process, ensuring the timely initiation of the study protocol for each participant. Upon randomization to the intervention arm, participants received a comprehensive 10-minute face-to-face physiotherapy session within the ward during the first day of their hospital stay.

The inclusion criteria include women who had given informed written consent for Category “4” CS due to fetal and maternal indications. Mothers who had undergone two or more CSs or abdominal surgeries, who had complications such as Diabetes Mellitus, Systemic Lupus Erythematosus, connective tissue disorders, sepsis, patients on Disease Modifying Anti Rheumatic Drugs (DMARDs) and oral steroids, patients who could not comply with the physiotherapy interventions such as mentally incapacitated patients, patients who were in the Intensive Care Unit (ICU), deliveries with operative complications, CSs with general/epidural anaesthesia and using patient-controlled anaesthesia, and patients with an abdominal hernia and Diastasis Rectus Abdominis (DRA) larger than 2 cm were excluded from the study.

### Intervention

#### Intervention group

In this RCT, one arm had received standard nursing care and the other arm had received a 10 min structured physiotherapy education program pre-operatively by a qualified physiotherapist (K.W.). During this session, a PowerPoint presentation was used to educate the participants, which was reinforced by a patient information leaflet pre-operatively. The participants were given an information leaflet with details of the exercise prescription with a pictorial representation of the exercises to be carried out, the number of repetitions to be carried out, the frequency of each exercise, physiotherapy education guidelines, and precautions to be taken when carrying out the exercises. All exercises and education guidelines adopted were evidence-based and described well in our prior RCT [1]. Any clarifications regarding prescribed exercises were addressed. Reinforcement about exercise and education guidelines was carried out twice per day by the research team. Feedback was also provided during the post-operative period by the research team to ensure adherence and treatment fidelity following the CS. Physiotherapy sessions were held in a private room to minimize potential bias in the care that post-CS patients received; thus, nurses caring for post-partum women were unaware of the identity of patients in the intervention or control groups. Therefore, both groups received standard nursing care.

#### Control group

The control group was only given the standard nursing care, which did not include any physiotherapy training or educational sessions, and was followed up during this period. Standard nursing care included advising women to mobilize after 12 h, removing the catheter after 12 h, administering analgesics, and monitoring vital signs in accordance with the prescribed instructions provided by the surgical team.

### Outcomes

Baseline data such as age, weight, height and parity and Body Mass Index (BMI) (reported at booking visit) were obtained from the patient’s clinical records. A Short Form Health Survey (SF-36) questionnaire that was validated for Sinhala and Tamil in Sri Lanka was employed in this study to assess post-natal QOL after six weeks following CS [20]. The questionnaire was handed over to each participant in a self-addressed envelope on the day of discharge to assess the quality of post-natal life after six weeks following CS. The SF-36 had eight scaled scores; vitality, physical functioning, bodily pain, general health perceptions, physical role functioning, emotional role functioning, social role functioning and mental health. The scores were weighted sums of the questions in each section. Scores range from 0 to 100.

A follow-up call was given by the principal investigator during and at six weeks to ensure that each participant filled out the SF-36 questionnaire. The assistance of the public health midwife was sought in case of failure to respond to the questionnaire.

### Sample size

Due to the lack of studies investigating the impact of face-to-face physiotherapy on post-natal QOL using the SF- 36 questionnaire following CS, this was conducted for two-independent study samples with a continuous endpoint. The probability of type I error-alpha was set at 0.05 and the power at 80% [21]. Thus, the calculated sample size was 23 mothers in each arm with a 1:1 ratio. To account for potential drop-outs, an additional 15% was added to the sample size. As a result, the final sample size in each group consisted of 27 women who were scheduled for elective CS.

### Randomization

Using a simple randomization design, pregnant women scheduled for elective CS were randomly assigned to one of two groups: the intervention group and the control group. A computer-generated random number sequence was placed within opaque and sealed envelopes and used to determine the allocation of participants to each arm upon recruitment to the study to reduce bias in the allocation of participants to the two arms of the study.

### Blinding

This was a single-blind RCT in which participants were blinded from definitive intervention. Hence, there was no attempt made to conceal the identity of each group from the investigators. The same physiotherapist carried out the pre-operative interventions and collected the outcome measurements.

### Statistical methods

Data obtained from the two groups were analyzed using Statistical Package for the Social Sciences (SPSS 20.0). The significance level was set at 0.05 and calculated at a 95% confidence interval. Descriptive statistics were utilized to analyze demographic details between the intervention and the control groups. Independent samples t-test was used to compare the post-natal QOL after six weeks following CS between the two groups.

## Results

### Participant flow

**Fig. 2:**
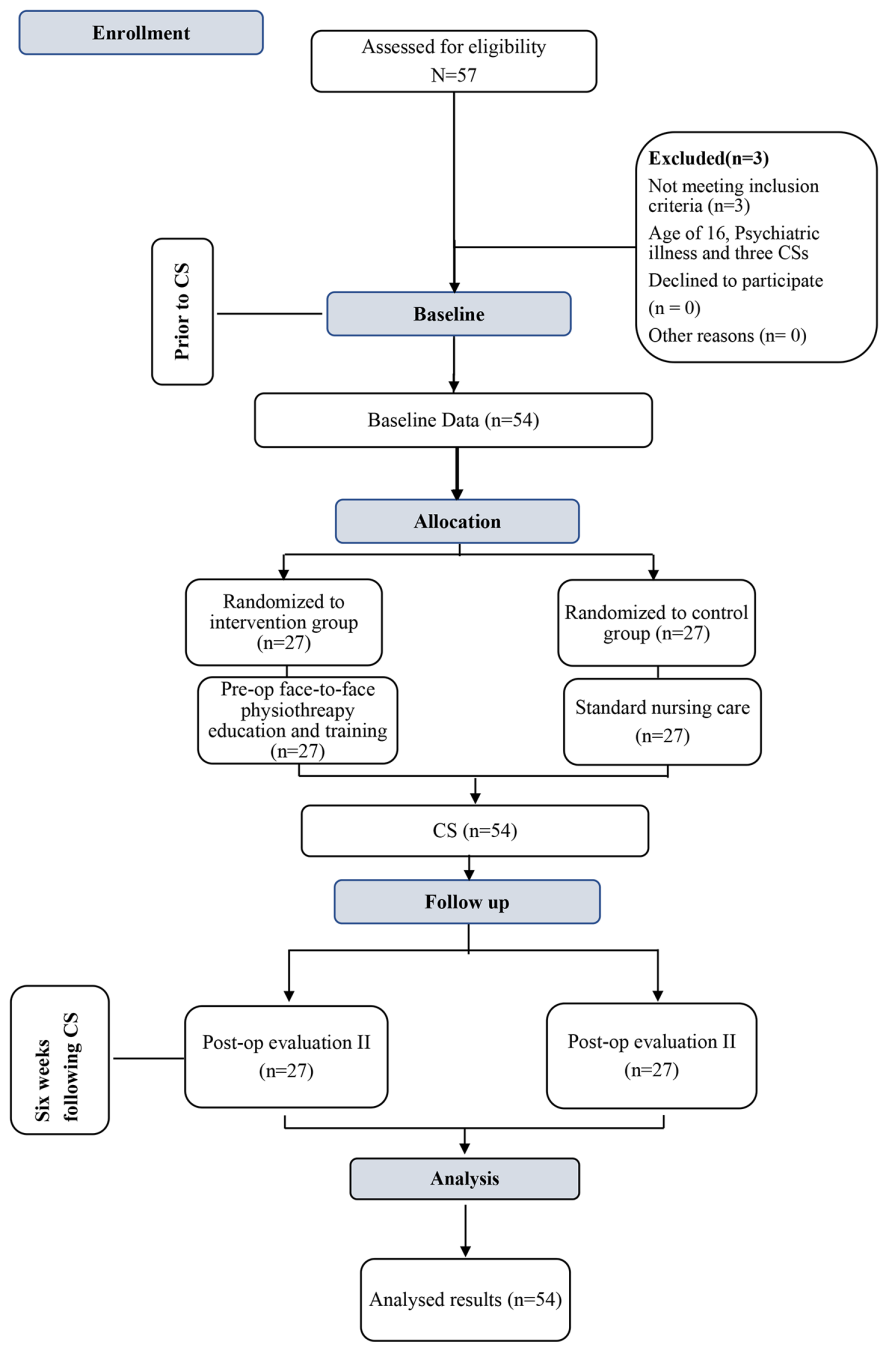
Flow chart of participants

All participants (n = 54; intervention arm = 27; control arm = 27) followed the intervention protocol actively and engaged in all the required measures from the initiation to the conclusion of the study. Since there were no drop-outs after recruitment, the study’s final analysis included every participant who had been recruited for it. The flow of participants through the study is shown in Fig. 2.

### Baseline data

Demographic information such as age, body weight, height, BMI at the booking visit, and parity were collected on the day of recruitment, which coincided with the first day of hospital admission. The characteristics of the study population are presented below [Table 1].

**Table 1:** Information on socio-demographic characteristics and descriptive data on anthropometry of the study population

Characteristic	Intervention group Mean ± SD	Control group Mean ± SD
Age (years)	29.96 (± 4.89)	32.63 (± 4.43)
Height (m)	1.56 (± 0.05)	1.57 (± 0.05)
Body weight (kg)	79.03 (± 11.72)	77.33 (± 9.57)
BMI at the booking visit (kg/m^2^)	32.63 (± 4.43)	31.55 (± 4.70)
Parity	1–2 (± 0.44)	2–3 (± 0.39)

As indicated in the table above, this trial comprised a population of young adults, with mean ages around 30 and 33 in the intervention and control groups, respectively. The estimated range for parity in the intervention group would be approximately 1 to 2. The parity range in the control group is estimated to be around 2 to 3.

### Outcomes and estimation

Mean SF-36 scores obtained by the study participants of the intervention and control groups under the eight scales of physical function, role limitation due to physical health, role limitation due to emotional problems, energy/ fatigue, emotional wellbeing, social functioning, pain and general health, were compared and the results are shown in Table 2.

**Table 2:** Comparison of the mean SF-36 score between the intervention and control groups

	Intervention group Mean ± SD	Control group Mean ± SD	Mean difference	p-value	95% confidence intervals
1. Physical function	94.1 ± 16.9	60.9 ± 21.1	33.2	< 0.05	52.7 to 73.6
2. Role limitations due to physical health	88.7 ± 10.3	51.3 ± 3.2	37.4	< 0.05	81.6 to 73.1
3. Role limitations due to emotional problems	42.5 ± 21.1	40.8 ± 18.3	1.7	0.09	36.5 to 38.8
4. Energy/ fatigue	67.6 ± 19.9	45.2 ± 15.8	22.4	< 0.05	35.5 to 54.9
5.Emotional well-being	44.0 ± 15.2	45.3 ± 13.7	-1.3	0.67	42.2 to 43.1
6. Social functioning	45.9 ± 6.5	45.7 ± 6.5	0.2	0.98	40.9 to 44.8
7. Pain	79.1 ± 5.3	53.9 ± 3.3	25.2	< 0.05	62.6 to 77.5
8. General health	80.4 ± 44.4	80.9 ± 44.8	-0.5	0.99	75.7 to 85.5

Participants in the intervention group demonstrated significantly higher mean SF-36 scores only for physical function, role limitation due to physical health, energy/fatigue, and pain compared to the control group. Importantly, no adverse events were observed.

## Discussion

This trial is a secondary analysis of the RCT that was conducted to investigate the effectiveness of face-to-face physiotherapy education and training on post-operative outcomes of women following CS, such as post-operative pain, doses of additional analgesics required, pain upon returning to functional activities, and lengths of hospital stay [1]. Through this secondary analysis of the above-mentioned RCT, we assessed the impact of physiotherapy training and education to enhance post-natal QOL after six weeks following CS. Improved post-natal QOL was shown in the domains of physical function, role limitation due to physical health, energy/fatigue, and pain in the intervention group who received structured physiotherapy education and training vs. the control group who did not receive such training.

Other studies conducted similarly have shown that post-natal physiotherapy exercises given during the immediate post-partum period help improve the QOL by enhancing physical, and general well-being [7, 12]. This is possible by improving body mechanics, reducing pain and improving blood circulation [8, 22]. Pre-operative or pre-emptive physiotherapy education aids in changing patients’ knowledge and behaviour, which influences all the domains of health-related QOL [23]. Another study has shown that simple, low-risk pre-operative education sessions and breathing exercises by physiotherapists within 6 weeks of upper abdominal surgery reduced post-operative pulmonary complications incidence. In addition, they concluded that pre-operative physiotherapy education sessions within 6 weeks of surgery can be utilized to empower patients to reach desired post-operative outcomes [24]. Pre-emptive psycho-education can help patients have accurate expectations, and decrease their anxiety levels and post-operative pain [25].

In the current trial, we implemented a 10-minute face-to-face structured physiotherapy education program as the intervention prior to the elective CS. Here, the primary focus was to enhance physical health-related outcomes of post-natal QOL including bodily pain, energy levels, fatigue, and physical symptoms such as post-partum urinary incontinence and low back pain [26] that would ultimately enhance the post-operative recovery following CS. It is important to note that QOL is a multidimensional construct encompassing broader domains, including physical, emotional, social, and overall health [27]. While our intervention was specifically designed to improve domains associated with physical well-being, we acknowledge that it may not directly address the emotional and social aspects of QOL. Emotional well-being and social functioning are influenced by various factors beyond the scope of the current physical therapy intervention, such as social support, psychological factors, and overall lifestyle [28]. This state of complete physical, mental, and social well-being contributes to overall health and well-being [29]. These factors may have contributed to the absence of significant differences in the mentioned domains, namely role limitations due to emotional problems, emotional well-being, social functioning, and general health, between the intervention and control groups.

To the best of our knowledge, this is the first RCT conducted in an LMIC to assess the effectiveness of physiotherapy training and education prior to elective CS to improve post-natal QOL. In the context of the current economic crisis, this simple and practical approach may assist in reducing the burden on the healthcare system in addition to improving other outcomes.

Health authorities should take the initiative to include physiotherapy education and training in their post-natal care protocols in all settings. Cost-effectiveness, staff allocation, and other logistics should be explored to make pre-operative and post-operative physiotherapy programs sustainable in LMIC settings.

### Limitations and recommendations

Due to the necessity of study participants continuing the post-operative physiotherapy exercise prescription at home after discharge, we were unable to directly oversee and ensure compliance with the physiotherapy protocol. Consequently, there is a potential reduction in patient adherence and treatment fidelity, which should be considered as a limitation. Additionally, it is acknowledged that important demographic measures were only collected at baseline, and there was a disparity in parity between the two groups. Moreover, it is important to note that the assessment of QOL was solely reported post-intervention. Therefore, it was not possible to compare the various domains of QOL before and after the physiotherapy session within the two groups. These factors introduce the possibility of bias in the results and should be taken into account.

This secondary analysis did not specifically assess the impact of physical therapy on pre- and post-natal QOL due to the absence of baseline data collection among the subjects. Consequently, it is imperative to conduct a more comprehensive study with a wider scope that not only evaluates the impact on the intervention and control groups but also both QOL at the beginning and end of the intervention. Simultaneously, existing research evidence suggests that interventions focusing on emotional and social well-being have shown effectiveness in improving these specific domains of QOL, ultimately enhancing overall health and well-being. Therefore, we propose that future studies investigate interventions that address a broader range of QOL dimensions, including emotional and social components, that could be integrated into the physical therapy protocols to gain a more comprehensive understanding of them.

While the current study provides valuable insights into an underexplored area of post-natal physiotherapy, it is essential to acknowledge that parts of the data related to the methodology in this manuscript have been previously published elsewhere [1]. Although our key objective in this study was to provide a comprehensive secondary analysis of the long-term follow-up data related to the post-natal QOL outcomes of the physiotherapy intervention, we recognize that some readers may be aware of the previously published data [1].

This endeavor enhances the informative value of the intervention package, highlighting the positive impact of this intervention on short-term post-operative outcomes, including reduced post-operative pain, the requirement for additional analgesics, pain upon returning to functional activities, and shorter hospital stays, while also underscoring its significant role in enhancing the physical health-related domains of QOL following CS.

Finally, future research on this topic of interest can be further advanced by including a few additional questions aimed at assessing the exercise adherence and treatment fidelity of the subjects. This inclusion would significantly contribute to the meaningfulness and comprehensiveness of the study.

## Conclusion

The results of this RCT suggest that physiotherapy training and education prior to elective CS are effective in enhancing the post-natal QOL in physical health-related aspects.

## Data Availability

Not applicable.

## **List of abbreviations**

ACOG: American College of Obstetricians and Gynaecologists
BMI: Body Mass Index
CS: Caesarean Section
DSHW: De Soysa Hospital for Women
LMIC: Low-and Middle-Income Countries
QOL: Quality Of Life
RCT: Randomized Controlled Trial
SD: Standard Deviation
SF-36: Short form-36
